# Multi‐'omic characterisation of vulnerable synaptic components in Alzheimer's Disease

**DOI:** 10.1002/alz70855_099033

**Published:** 2025-12-23

**Authors:** Tatiana Georgiades, Eileen A Chandra, Bshaier Allehyany, Eleonore Schneegans, To Ka Dorcas Cheung, Harry Whitwell, Samuel J Barnes, Paul M Matthews, Johanna S Jackson

**Affiliations:** ^1^ Imperial College London, London, London, United Kingdom; ^2^ Imperial College London, London, Greater London, United Kingdom; ^3^ Dementia Research Institute at Imperial College, LONDON, United Kingdom; ^4^ Imperial College, London, United Kingdom; ^5^ UK Dementia Research Institute at Imperial College, LONDON, London, United Kingdom

## Abstract

**Background:**

Synapse loss is the strongest pathophysiological correlate of cognitive decline in Alzheimer's disease (AD). Untargeted synaptic proteomics in *post‐mortem* human brain has shown a downregulation of synaptic transmission pathways and a progressive increase in pathways related to synaptic vulnerability in regions with increasing AD pathology. Evidence from bulk tissue transcriptomics has highlighted changes in the levels of synaptic genes in AD. However, the local synaptic transcriptome has been shown to be essential for synaptic plasticity and cognitive function. Here, we integrated proteomic and transcriptomic data from isolated synaptoneurosomes to comprehensively characterize the molecular changes occurring in mid‐stage AD.

**Method:**

We used post‐mortem human tissue from the middle temporal gyrus of 33 AD cases with moderate (Braak III‐IV) pathology, alongside 33 non‐diseased controls (NDC, Braak 0‐II), to isolate synaptoneurosomes via the SynPER (Synaptic Protein Extraction Reagent) preparation method. Label‐free quantitative proteomics using LC‐MS/MS and bulk RNA‐seq was conducted on the synaptoneurosome isolations. Multi‐‘omic data integration was performed using *Omix* v1.0.0 on R. Histopathological characterization of β‐Amyloid and pTau (PHF1) was performed on the same cases using immunohistochemistry.

**Result:**

Preliminary differential protein expression analysis demonstrated downregulated mitochondrial function in mid‐stage AD. Functional enrichment using SynGO, showed higher involvement of the pre‐synaptic active zone in AD compared to NDC. Nonetheless, there were no notable differences in the overall involvement of the pre‐synaptic compared to the post‐synaptic compartment. Differential expression analysis of proteomic and transcriptomic layers enabled us to determine the role of local protein synthesis in synaptic protein expression. The contribution of amyloid and tau pathology to the variation observed across omics layers was determined using Multi‐Omics Factor Analysis.

**Conclusion:**

Collectively, our results elucidate the changes in the synaptic proteome preceding widespread synapse loss in AD, along with their potential underlying mechanisms, thus providing targets for early therapeutic interventions.

